# Hilde Mangold-Pröscholdt (1898 – 1924): The Spemann-Mangold Organizer

**Published:** 2016-03-28

**Authors:** J VAN ROBAYS

**Affiliations:** Department of Pathology, ZOL, Campus St Jan, Schiepse Bos 6, 3600 Genk, Belgium.

**Figure:**
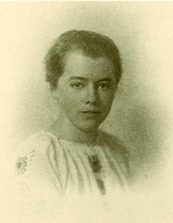


In the history of medicine, few women were awarded an eponym. One can assume that this doesn’t have anything to do with their second X-chromosome or the weight of their brains. Most probably, it was the era that was not on their side. In the time period wherein most eponyms were given, women had almost no access to higher education. There are a few exceptions of course, such as Marie Curie or Virginia Apgar. But there have also been less famous women who conducted groundbreaking scientific research. Hilde Pröscholdt is an example thereof. It’s a shame her life ended so prematurely and tragically.

## Hilde Pröscholdt

Hilde was born on the 20th of October, 1898, in Gotha, Thüringen. She was the second of three daughters of Ernst and Gertrude Pröscholdt. Her father began as a handicraft porcelain painter but became a clerk in a soap factory after moving to Gotha. An ambitious man, he quickly rose to higher executive positions and was allowed to marry the daughter of the owner. Hilde’s mother was widely culturally interested and was also active in politics, where she defended women’s rights. In the spirit of the German Bildungsbürgertum, Hilde received the very best of educations. After high school, she was enrolled at the age of 16 at the Gymnasium Ernestinum, in those days almost inaccessible to girls. After obtaining her degree with excellent grades, her parents didn’t send her off to college. She had to go to a boarding school where girls learned housekeeping and social etiquette. She didn’t last long at that girl’s boarding school though. Six months later, she was enrolled at the university of Jena as a chemistry student. But chemistry wasn’t really her cup of tea either. She was more passionate about biology and zoology. So she went to Frankfurt am Main to pursue those interests.

**Fig. 1:**
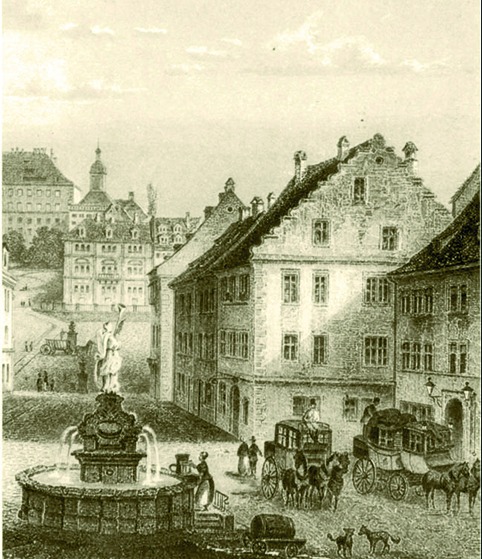
— City of Gotha: market place

## Hans Spemann

Around 1900, very little was known about embryogenesis. How does a complex being develop from a simple, round egg? Where in this developing embryo lies the information that regulates its construction? Is it in every cell, or only in well-defined groups of cells? And how do the dividing embryonal cells know whether to build a leg, a head or a tail? By the year 1900, these were towering questions. To find an answer, the German embryologist Hans Spemann started to do experiments on salamander eggs from 1903 on. Those were easy to get and big enough to work with. Being an experimenter, Spemann was very resourceful in assembling his own surgical arsenal. For example, he cut a tress of his daughter’s hair and made miniature lassos out of it (Fig. 3). Because baby hair is soft, thin and flexible, this was the ideal tool to pinch a cluster of dividing salamander cells. That way, he observed that from two pinched halves, two salamanders originated, both of them with a normal build and perfectly viable. So, out of one fertilized egg, more than one individual could be generated, as we know is the case with identical twins.

**Fig. 2:**
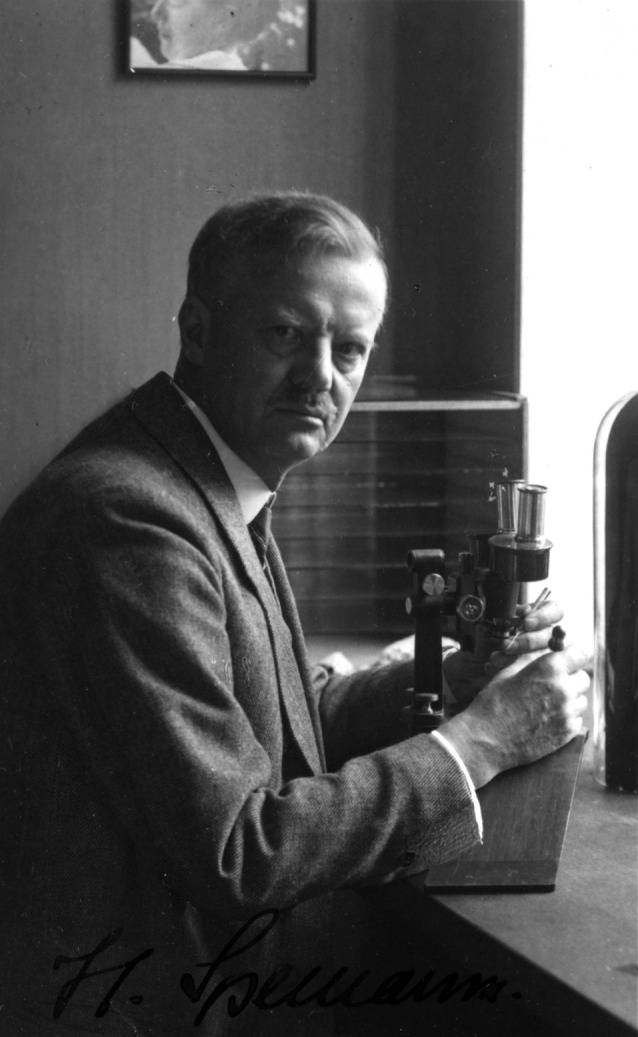
— Hans Spemann: German embryologist

**Fig. 3:**
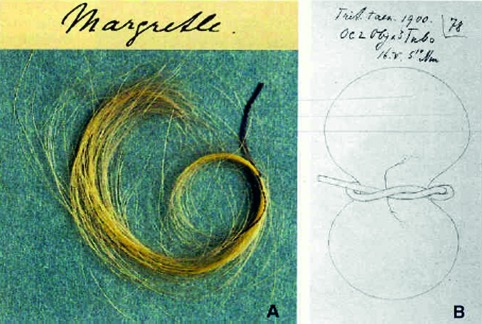
— Tress of Spemann’s daughter hair to make miniature lassos

## Meeting Spemann – Hilde

One day, Hans Spemann came to Frankfurt and gave a lecture about embryology. We discussed his recent discoveries in this young, “embryological” domain of Zoology. After his exposition, Hilde was so begeisterd that she asked the professor if she could do a PhD at his institution. This was allowed, and so she went to Freiburg, where Spemann was the head of the department of Zoology. For two semesters, Hilde studied this brand-new trade and gained some practical laboratory experience. In the lab she was mentored by Fritz Baltzer, Bruno Geinitz and Otto Mangold. She fell in love with the latter – or he with her – and they got married on her 23^rd^ birthday. Otto Mangold was Spemann’s favourite assistant and his right hand, but soon Spemann came to appreciate Hilde’s capabilities as well. As the head of his department, he was overwhelmed by all kinds of administrative tasks and was no longer able to conduct his experiments himself, so he handed them down to this “power couple”. Otto was an enthusiastic scientist and a hard worker. With her dexterity (taught by sewing lessons at the boarding school) , Hilde was ultimately suited to execute minuscule surgical operations. She began her experiments in 1921 and made use of the microsurgical instruments Spemann had invented. Besides the already mentioned mini-lassos made out of baby hair, she also made use of incredibly thin glass needles, often heated, to cut certain parts from the embryos or to burn them away. To fix the eggs in one placewhile performing these operations, Spemann had also designed wax plates with tiny wells. Spemann is sometimes referred to as the father of microsurgery, and there’s something to be said for that.

## The Spemann – Mangold organizer

**Fig. 4:**
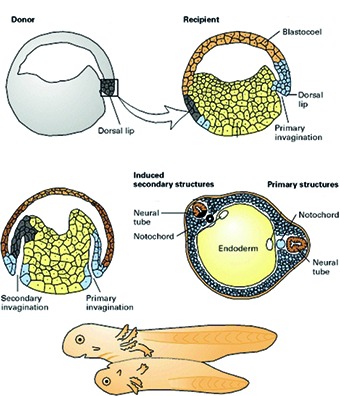
— Dorsal lip transplantation in a salamander embryo

In the specific developmental stage Hilde was working with, a salamander egg is a tiny globule of only 1.5 mm in diameter. For her PhD thesis, she picked a minuscule tissue sample out of the egg, not even the size of a pinprick, and inoculated it onto another salamander embryo (Fig. 4). She didn’t just take a random piece of tissue. She took a very small region, appointed by Spemann, which he believed to be the location of those cells that were forming the major part of the three germ layers. Embryologists call it “the dorsal lip”. It is situated at the dorsal side of the developing embryo. According to Spemann, this dorsal lip developed further into the neural plate, the notochord and the somites. Simply put: a body axis with spine, spinal cord, brain, head and tail. Hilde Mangold performed these transplantations so skillfully that the embryos not just survived but continued their development. And the result was spectacular. From the piece of dorsal lip that she had transplanted from the dorsal side of one embryo onto the ventral side of another, a new body axis developed, a second spine with all the accessory structures in place. This way a second salamander originated, grown belly to belly onto the first one. Quite a monstrous Siamese twin maybe, but from the experiment an important conclusion could be drawn: that little piece of dorsal lip had organizational capacities. It was able to steer the adjacent cells, which up to that point didn’t have a “destination” yet, towards organization into a second body axis. Spemann therefore named this region “the organizer”.

After 259 transplantations, Hilde concluded her experiments in February 1923 and wrote her doctoral thesis. Because Spemann sensed that this wasn’t a simple matter, and would sound quite new to the older professors in the jury, he added a cover letter to her thesis. Herein he shortly summarized the experiments and gave some comments on the results. At the end of his letter he praised the work of his assistant: "The great technical difficulties that arose with the transplantations were easily overcome by Mrs. Hilde Mangold, thanks to her dexterity and perseverance.” Because he also sensed that the discovery of the “organizer” would shed a completely new light on embryogenesis, he concluded his letter with: “The positive results of these experiments are of great theoretic importance.” And Spemann was right. The discovery of the “organizer” was the go-ahead to resolve the mysteries surrounding the structural development of the embryo. He was later awarded for it with the Nobel price.

## Tragedy

**Fig. 5:**
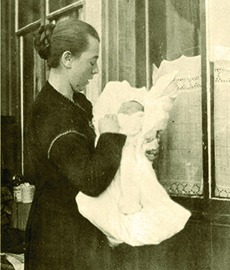
— Hilde and son Christian

In December 1923, Hilde gave birth to a son, Christian (Fig. 5). A few months before, Otto had accepted an invitation to become the head of the Department of Zology at the Kaiser-Wilhelm Institute in Berlin-Dalhem. Otto was over the moon, and so was Hilde. To her sister, she wrote "I’m incredibly happy for Otto. It really is a beautiful and independent position. Maybe Otto is still a bit young, and Spemann would prefer to keep him close for a couple more years, but it’s such a wonderful opportunity that we don’t have any other choice.” Less than a year later, she was dead. In a letter to Fritz Baltzer, Spemann narrates the tragic events. While visiting her parents in law, Hilde tried to light a petrol stove to heat up her son’s meal. She was refilling the stove when she spilled a bit of petrol and in no time, everything was lit up in flames. Like a burning torch she ran outside, but neither her mother in law nor Otto were able to do anything for her anymore. She passed away the next morning. She hadn’t even reached the age of 26 years.

## Nobel Prize in Medicine 1935

In sharp contrast to her premature death stands her life work, which is of perpetual value. With her doctoral thesis, still awaiting publication at the moment of her demise, she and her mentor Hans Spemann had laid down the foundations of what would later be called the “embryonal induction”. This process describes how a particular small group of cells in a developing embryo is capable of programming adjacent cells to organize themselves into well-defined anatomical structures. Structures from which, ultimately, a recognizable, living creature originates, with its head, nose, ears and eyes in the right place. And a tail at the end. For his discovery of the "organizer", Hans Spemann received the Nobel Prize in 1935. Deservedly! The “Spemann-Mangold organizer” was the “Rosetta Stone" of Embryology, finally enabling the decryption of the code for the embryo’s developmental processes. It still took several decades before scientists gained some insight into the mechanism that was active in this organizer. How could such a small piece of tissue be able to program the surrounding cells? And in which form was this information packed? It was not until the discovery of the DNA structure that it became clear that this double helix was the carrier of the genetic material that controls all of life’s processes, including the blue print of an embryo. Up until this day, the Spemann-Mangold organizer is a gold mine for geneticists. Day after day, they continue to discover new genes and derived proteins in it, which, if turned on and off at the right time, ultimately realize the incredibly complex building plan for an embryo.

## Epilogue

**Fig. 6:**
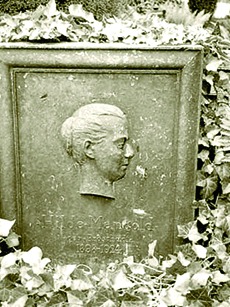
— 'Tomb’ Hilde Mangold

Hilde Mangold was not present at the awards ceremony in Oslo. She was buried 11 years earlier in the Gotha cemetery, beneath a bronze memorial plaque designed by a befriended artist from Freiburg, Julius Bissier (Fig. 6). A Nobel prize is never awarded to a deceased individual. Nevertheless, Hilde Mangold would have deserved one. It would have been the first one, and until this day the only one, ever to have been based on a doctoral thesis of an assistant.

